# Clinical Manifestation, Histopathology, and Imaging of Traumatic Injuries Caused by Brazilian Porcupine (*Sphiggurus villosus*) Quills

**DOI:** 10.1155/2016/7851986

**Published:** 2016-11-17

**Authors:** Lívia M. Araújo Jorge, Fred Bernardes Filho, Fabrício Lamy, Laila Klotz A. Balassiano, Loan Towersey, Roderick Hay, Marco Andrey C. Frade

**Affiliations:** ^1^Dermatology Division, Policlínica Geral do Rio de Janeiro, Rio de Janeiro, RJ, Brazil; ^2^Dermatology Division, Department of Internal Medicine, Ribeirão Preto Medical School, University of São Paulo, Ribeirão Preto, SP, Brazil; ^3^AIDS Division, Carlos Tortelly Municipal Hospital, Ministry of Health, Niterói, RJ, Brazil; ^4^Kings College NHS Hospital Trust, London, UK

## Abstract

Injuries to humans caused by porcupines are rare. However, they may occur due to the proximity of urban areas and the animal's habitat in areas such as the Floresta da Tijuca in Rio de Janeiro. Outdoor sports and leisure activities in areas close to forests or in the rain forest are also relevant for incidents of this kind and a better knowledge of the local forest fauna would prevent such undesirable accidents. Porcupine quills have microscopic barbs at their tips which facilitate skin penetration, but hampering their removal. Once the spines are lodged in tissue, the microscopic backward-facing deployable barbs at the tips cause trauma if anyone tries to remove them. Local haemorrhage and an inflammatory response to the contaminated foreign body occur. Depending on the time lapse in removing the spines either septic or sterile foreign body reactions may occur. There is also the risk of migration of the spines, where fatal cases have been reported in human and veterinary medicine. Herein we report two unusual cases of accidents involving humans and the South American porcupine. The* Sphiggurus villosus* spines removed from scalp skin were also documented through Scanning Electron Microscopy.

## 1. Introduction

Rodents of the class, Erethizontidae, include New World porcupines, commonly known as “ouriços-cacheiros” or South American porcupines in Brazil. They are distributed across three genera:* Coendou*,* Sphiggurus,* and* Chaetomys* [1]. A comprehensive taxonomic revision of the genus* Coendu* may shed further light on species limits and the geographic ranges of* C. prehensilis* and other congeneric forms [2].* Sphiggurus villosus* is the most commonly identified porcupine in the Rio de Janeiro area.* S. villosus* Cuvier (1822) is the third currently recognized Atlantic Forest form. The specimens originally described by Cuvier were collected by A. de Saint-Hilaire, who made several excursions in Rio de Janeiro and Minas Gerais states [2]. The species is present in Atlantic Forest habitat, it occurs in primary forest but is more common in secondary forest and forest borders, and it is occasionally found near urban areas.* S. villosus* showed a similar autosome complement (chromosome morphology and basic number) as that described for* S. vestitus pruinosus* [2, 3]. South American porcupines are arboreal animals with crepuscular and nocturnal habits [4–6]. They have short, stiff, and sharp spines completely or partially covered by long hairs. The following three patterns of spine bands have been observed in Brazilian porcupines in the wild: bicoloured spines (white or whitish-yellow base and black tip); tricoloured spines (white or whitish-yellow base, blackish middle, and whitish tip); and four-banded spines (identical to the tricoloured spine with an additional brownish tip) [4, 7].

Once spines are lodged within tissue, they are difficult to remove. This is due to the microscopic backward-facing deployable barbs that are present at their tips. Herein we report two accidents involving humans and South American porcupines and used Scanning Electron Microscopy to show the geometry of Brazilian porcupine spines and their tip surface after they have been removed from the skin.

## 2. Case  1

**Figure 1:**
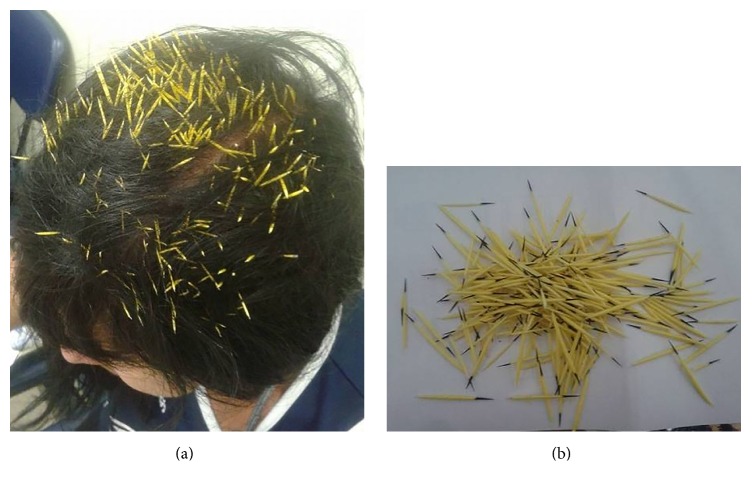
(a) Patient's 1 scalp one covered with many porcupine spines; (b) Brazilian porcupine spines removed from patient 1.

A 52-year-old female patient was walking in the Gávea neighborhood, Rio de Janeiro, Brazil, when a porcupine dropped from a lamp post onto her head. Due to intense pain, she was admitted to the emergency department. After cleansing of the area with chlorhexidine and alcohol 70%, about 200 black-tipped yellow spines were removed from her head with tweezers with no anaesthesia (Figure 1). She was medicated with intravenous tramadol 100 mg. The spines were typical of* S. villosus* species (orange-spined hairy dwarf porcupine).

**Figure 2:**
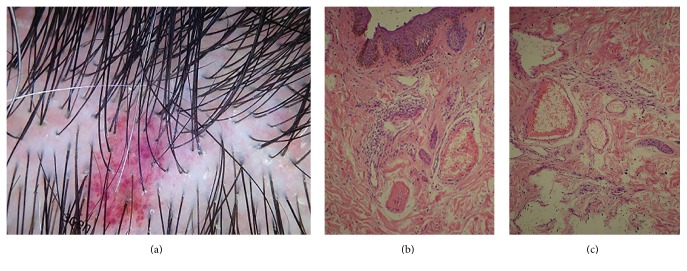
(a) Dermoscopy of a reddish-purplish lesion showing red and violet globules; (b-c) irregular acanthosis with ectasia and superficial venules congestion with superficial perivascular mononuclear infiltrate (hematoxylin and eosin staining; (b) 40x, (c) 100x).

Seven days later, the patient was seen in a dermatology consultation complaining of local pain. She believed there were residual fragments of spines on her scalp. On physical examination, remaining fragments were not found, but reddish-purplish lesions were observed. Dermoscopy showed red and violet globules. The histopathological examination of a lesion showed irregular acanthosis with ectasia and superficial congested venules with a superficial perivascular mononuclear infiltrate (Figure 2). The patient was medicated with diclofenac sodium, 50 mg orally three times a day, for five days. Fourteen days after the accident, she did not have any pain, fully recovering from her injuries.

## 3. Case  2

**Figure 3:**
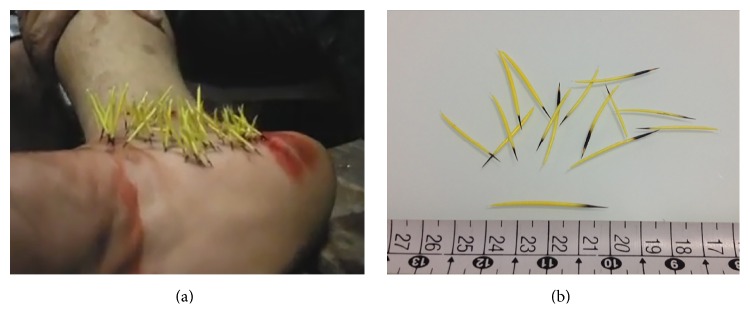
(a) Medial aspect of right foot covered by dozens black-tipped yellow spines; (b) Brazilian porcupine spines, measuring about 4 cm.

A 20-year-old male patient was returning from his work in Barra de São Francisco, Espírito Santo, Brazil, riding a motorcycle. On the way home, in a rural area in the outskirts of this small town, he observed a small animal crossing the track in the dusk which he believed to be an armadillo. Fearing that it might cause an accident, he kicked it with his right foot. He was wearing slippers and felt intense pain, observing that there were numerous porcupine spines on the medial side of his foot. He stopped at the closest place where he could get aid, a local bar. The injuries were washed with alcohol and about 100 black-tipped yellow spines were removed from his foot with tweezers. Later, at Dra Rita de Cassia Hospital, he was medicated with intravenous ketoprofen 100 mg. The spines were typical of* S. villosus* species (Figure 3). He was also given diclofenac sodium, 50 mg orally three times a day, for seven days. He did not have any complications and returned to his normal activities eight days after the accident.

## 4. Discussion

**Figure 4:**
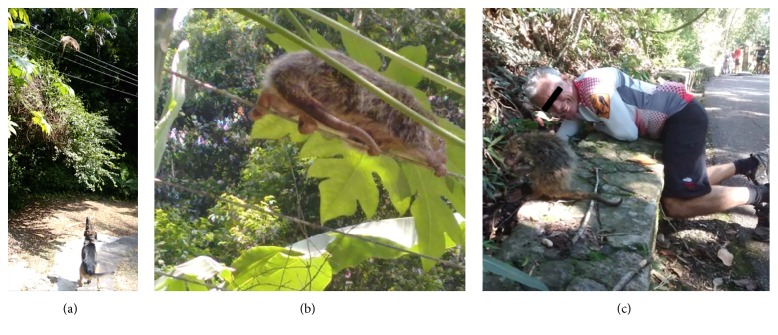
(a) Altercations between porcupine and dog in periurban areas; (b)* Sphiggurus villosus*, Brazilian porcupine; (c) interaction between cyclists and a porcupine during track cycling in the Tijuca forest.

*S. villosus* spends most time up in the trees in the rain forest, only coming down to search for food or to defecate. The orange-spined hairy dwarf porcupine,* S. villosus*, is a porcupine species endemic to southeast Brazil with a prehensile tail (Figure 4). In this region, there has been vast degradation of their habitat, the Atlantic rain forest. In rural areas, close to original or secondary patches of rain forest, porcupines may come down to the fields in search of food in the crops. Hunting of the species, although forbidden, does occur and accidents involving humans and hunting dogs may occur. Sometimes, the porcupine may search for food or shelter in residences, garages, or barns, and altercations between porcupines and dogs may occur (Figure 4). Injuries from spines may cause severe lesions, sometimes with fatal consequences, due to migration of the spines or secondary infection [8].

Gávea district is adjacent to Tijuca National Park (Tijuca rain forest), the biggest urban forest in the world, located in the heart of cosmopolitan Rio de Janeiro. This reforested area (3,953 ha) of Atlantic rain forest harbours numerous species of animal. Accidents such as in case 1 occur due to the proximity of the urban area and the dense Atlantic tropical forest. Porcupines climb up trees and from these they may accidentally move on to wires or posts in residential areas close to the reforested areas. The urban inhabitants living close to the forest as well as tourists visiting the forest and people practicing sports (Figure 4(c)) may accidentally come upon the porcupine with a consequent risk of injuries from spines as these are part of the animal's defense mechanisms [5, 9]. Accidents like these are considered rare or anecdotal. Outdoor sports and leisure activities in the areas close to forests or in the rain forest and trails and roads that cross as well as dwellings bordering the forest increase the risk of these events.

Porcupines release their spines at will or when the skin is pressed, and small lances are discharged as a defense mechanism [4, 8]. These hard-to-remove spines are a biological adaptation of the porcupine providing a model for various piercing devices that need to be fixed after perforation. Once the spines are lodged in the tissue, the microscopic backward-facing deployable barbs that are present at the tips cause trauma if there is any attempt to remove them. Local haemorrhage and inflammatory response to the contaminated foreign body occur. Depending on the time lapse between injury and removing the spines septic or sterile foreign body reactions may occur. The kinetics of the trauma probably explains the duration of pain reported by both patients: in case 1, the free fall of a porcupine from a lamp post on the head of a female and in case 2, the kicking of a porcupine while riding a motorcycle. The reddish-purplish lesions with red and violet globules at dermoscopy observed on patient's 1 scalp are consistent with the histopathological findings (Figure 2). The absence of foreign body granuloma on the histopathology and the characteristics of the inflammatory infiltrate are consistent with the prompt healing of the patient.

**Figure 5:**
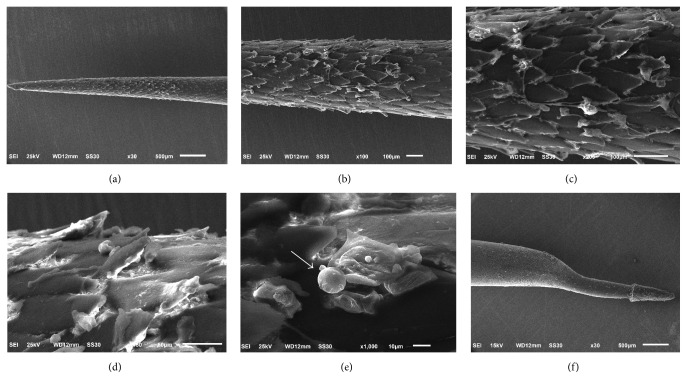
Scanning Electron Microscopy (JEOL JSM-6610LV) of the Brazilian porcupine spine; (a) distal tip enlarged 30x with fish scale appearance; (b-c) the spine enlarged 100x and 200x, respectively, showing liftable barbs covered with tissue debris; (d) in particular, lifted barbs enlarged 450x with tissue debris; (e) 1000x, red blood cell also observed on the surface of the spine (arrow); (f) 30x, the proximal end, which is attached in the porcupine, is covered by a hood and presents a curved shape.

A Scanning Electron Microscopic (SEM) examination (JEOL JSM-6610LV) of seven spines was performed. SEM revealed that the entire surface of quills had a fish scale appearance. The proximal end, which is attached to the porcupine, is covered by a hood and has a curved shape. It probably facilitates the elevation of the spine when the animal adopts a defensive position, through bristling. On the distal tip, the spine surface shows extendable barbs, which are responsible for locking onto the skin. These barbs resemble lances and they are covered with tissue debris; the presence of a red blood cell can be observed, which indicates that the quill punctured a dermal vessel or lacerated tissue upon removal (Figure 5).

The barbs at the tips of spines facilitate skin penetration and fixation, hampering their removal [4, 10, 11]. These barbs are elevated when the spine is pulled out, thus increasing injury. A study conducted by Cho et al. [11] showed that barbed spines required approximately half the penetration force of barbless spines—either those that are naturally barbless or those sanded clean—and only 56% of the force needed for a hypodermic needle to breach the skin. The ultrastructure of the spine allows for perfect perforation (puncturing) and firm anchoring to its target. This biological adaptation of the porcupine spine provides a natural model for various piercing devices that need to be fixed after perforation.

A common misconception is that if you cut the spine, it will deflate and be much easier to pull out. On the contrary, this procedure may impair the clamping of the spines and their extraction. If the spines are not removed from victim immediately, they absorb body fluid and expand, causing the barbs to flare farther outward. Thus, each movement of the victim's muscles or body helps an embedded spine to go deeper and/or migrate. Anecdotally, such migration has led to internal organ damage and even death in humans. The spines of the Asian, African, and European rodents are longer and can cause deep visceral lesions. The cases described here are caused by species that possess short spines. A human accident involving a porcupine, with a spine piercing the intestinal wall, resulting in fatal peritonitis has been reported [12]. Knowledge about porcupine accidents is mainly anecdotal in our country, but there are cases reported in both human and veterinary medicine in Brazil [4, 13]. The Brazilian Environmental Health Surveillance has a notification system for monitoring biological risk factors related to poisonous animals (snakes, scorpions, spiders, Hymenoptera, and Lepidoptera), which can result in accidents of interest to public health. However, there is no central data base in our country for accidents caused by porcupines, despite human and veterinary interest [14].

The reported cases help illustrate dermoscopic, histopathologic, and SEM aspects of impact aggravated porcupine spine injuries. Despite a benign evolution in both cases, the morphologic aspects of the spines involved in this kind of cutaneous accident potentially present severe risks. People should be warned not to mistakenly frighten, play with, or capture porcupines. Local environmental authority who have staff with appropriate training and equipment should be contacted to capture and return these animals to their habitats.
